# Particularities of the Post-Pandemic Hepatitis A Outbreak in a Tertiary Infectious Diseases Hospital in Romania

**DOI:** 10.3390/jcm14124368

**Published:** 2025-06-19

**Authors:** Georgiana Neagu, Violeta Molagic, Serban Nicolae Benea, Irina Ianache, Eliza Militaru, Iulia Nedelcu, Gabriel Maxim, Gabriela Andreea Dumitru, Cristiana Oprea, Ruxandra Moroti

**Affiliations:** 1 Clinical II Department, Carol Davila University of Medicine and Pharmacy, 020021 Bucharest, Romania; georgiana.neagu@rez.umfcd.ro (G.N.); violeta.molagic@umfcd.ro (V.M.); irina.ianache@umfcd.ro (I.I.); eliza.manea@drd.umfcd.ro (E.M.); iulia-maria.nedelcu@drd.umfcd.ro (I.N.); gabriel.maxim@rez.umfcd.ro (G.M.); gabriela-andreea.stoica@rez.umfcd.ro (G.A.D.); 2 National Institute for Infectious Diseases ‘Matei Bals’, 021105 Bucharest, Romania; 3 Babes Clinical Hospital, 030303 Bucharest, Romania

**Keywords:** hepatitis A, post-pandemic outbreak, hepatitis A severity, MSM, HIV, syphilis, sexually transmitted diseases, STD, hepatitis E coinfection

## Abstract

**Background/Objectives:** In addition to classical transmission routes, hepatitis A (HA) outbreaks were, for the first time, linked to sexual activity in the late 1970s, particularly among men who have sex with men (MSM). Since then, outbreaks have continued to emerge globally among adults engaging in high-risk sexual behaviors, reinforcing the ongoing public health significance of this transmission route. Although the COVID-19 pandemic temporarily reduced HA cases, outbreaks reemerged following the relaxation of pandemic measures. This study aims to report the HA outbreak registered at Romania’s tertiary infectious diseases hospital during the first half of 2022. **Methods:** We retrospectively analyzed all HA cases admitted to the National Institute for Infectious Diseases from 1 January to 1 August 2022. **Results:** Among 51 cases, eight children (under 14) were excluded from further analyses. Of the 43 remaining cases, 37 (86%) were male, with 20/37 (54.05%) identified as MSM. Twenty-two of the males (59.45%) were previously or concomitantly diagnosed with sexually transmitted diseases (STDs), notably syphilis and HIV. A marginal finding: 14 out of 29 patients screened for hepatitis E tested positive for IgM. The MSM patients exhibited significantly higher transaminase levels (ALT median level 3404 U/L [IQR 2608–5448] vs. 2066 U/L [IQR 1393–3682]) and more severe liver impairment (INR median level 1.37 [IQR 1.18–1.78] vs. 1.18 [IQR 1.11–1.43] compared to non-MSM patients. While direct bilirubin levels were higher in MSM (7.4 mg/dL vs. 5.5 mg/dL), the difference was not statistically significant. **Conclusions:** An HA outbreak occurred at the beginning of 2022 among Romanian young MSM, with a more severe acute liver injury. High-risk sexual practices may be a potential risk factor for severe HA. This emphasizes the need to screen for STDs in young males with HA and review vaccination policies for at-risk individuals.

## 1. Introduction

Hepatitis A virus (HAV) is a small, non-enveloped RNA virus of the *Picornaviridae* family. It is highly contagious due to its resistance to a great variety of physical and chemical agents and its easy route of transmission, the fecal–oral route [1,2]. In addition to the well-established fecal–oral route, person-to-person transmission is possible. This includes unprotected sexual contact and sharing drugs [3,4,5].

All immunologically naïve humans can become infected through ingestion of contaminated products or via direct contact with a person with hepatitis A, including high-risk sexual practices [6,7].

Hepatitis A is an acute, self-limited infectious disease with a mild-to-moderate course in most cases. Fulminant hepatitis A is very rare (0.3–0.6% overall, but up to 1.8% in adults aged more than 50 years old) with mortality that could reach 80% [8]. There is no chronic carriage. Immunity after illness or vaccination for hepatitis A is considered lifelong [8].

In the last decades, epidemiology has shifted in some parts of the world, as there have been multiple outbreaks associated with risky behaviors. The potential risk of acquiring hepatitis A through high-risk sexual behaviors was first recognized in the late 1970s, when a higher prevalence of hepatitis A was observed among men who have sex with men (MSM) compared to heterosexual men [9,10,11,12]. Since then, periodic outbreaks have occurred globally [13,14,15,16,17], with an increasing number of cases up until the COVID-19 pandemic. For example, the incidence of hepatitis A in the USA increased by 294% during 2016–2018 compared to the first half of the decade, and outbreaks were driven by infection among persons who use drugs, persons who are homeless, and MSM, respectively [5,18,19].

In their retrospective analysis of HAV cases admitted to Vienna General Hospital between 2008 and 2018, Bauer et al. (2021) [20] found that severe disease progression, including liver dysfunction, was more common among younger males (31.5 vs. 63 years, *p* < 0.001) compared to other hepatitis A cases. The study coincided with a multinational hepatitis A outbreak among MSM, leading the authors to propose that many young male patients in their cohort might also engage in sexually risky behaviors and suggesting that young males, particularly MSM, may be at greater risk for severe forms of hepatitis A [20]. Similar conclusions were reached in other studies [21,22,23,24].

In Western Europe, there is a low incidence of hepatitis A, and usually, new infections are linked to travel to endemic countries [20].

Romania has an intermediate endemicity for hepatitis A [25]; most cases are linked to poor hygiene standards, with viral acquisition via contaminated food and water and with outbreaks in collectivities and family clusters, predominantly in children.

In early 2022, we observed a sudden surge in hepatitis A cases at our tertiary care institute. Intriguingly, the cases predominantly affected young males rather than occurring primarily in children, as expected. The aim of our study is to characterize this outbreak following the lifting of precautionary measures at the end of the COVID-19 pandemic.

It is to be mentioned that the hepatitis A vaccine is not included as a routine vaccine in the Romanian national vaccination program.

## 2. Materials and Methods

There is a retrospective descriptive study. We analyzed all hepatitis A cases admitted in the first seven months of 2022 to the National Institute for Infectious Diseases ‘Prof Dr Matei Bals’ (INBI), Bucharest, one of Romania’s largest infectious diseases settings. INBI has several hundred beds for adults and children and is also the largest Romanian center for HIV/AIDS treatment and care.

The tests conducted to identify the viral cause of acute hepatitis include hepatitis A virus IgM antibodies (HAV-IgM), hepatitis B surface antigen (HBs-Ag), hepatitis B core IgM antibodies (HBc-IgM), hepatitis C virus antibodies (HCV-Ab), hepatitis D virus IgM antibodies, and hepatitis E virus IgM antibodies (HEV-IgM). Notably, HEV-IgM alone is insufficient for confirming acute hepatitis E. HEV-IgM must be accompanied by HEV-RNA or HEV-IgG antibodies for accurate diagnosis.

Hepatitis A diagnosis was suspected upon clinical and biological criteria and was confirmed through a positive HAV-IgM assay.

Age, gender, sexual orientation, the presence of sexually transmitted diseases (STDs), clinical examination, liver ultrasound, blood tests, including liver enzymes: alanine aminotransferase (ALT) and aspartate aminotransferase, direct and total bilirubin levels, and coagulation indices (prothrombin concentration and INR—International Normalized Ratio), period of hospitalization (days), and the outcome were assessed.

After analyzing the demographics of all hepatitis A cases admitted during the study period, we excluded children under the age of 14 from further in-depth analyses.

We stratified the cases according to severity and prognosis, as per the last guideline of the European Association for the Study of the Liver (EASL), 2017, including King’s College criteria regarding prognosis [26]:

Acute liver injury (ALI) is defined as a two-to-three-fold elevation of transaminases associated with jaundice and coagulopathy of primary liver etiology in a patient without preexisting chronic liver disease. By consensus, coagulation parameters commonly used are INR or its equivalent, prothrombin time (PT). It is considered an altered coagulation state or coagulopathy, an INR > 1.5 or an equivalent prolongation in PT. There are two other INR breakpoints, >3.5 and >6.5, which are considered for the poor prognosis of acute liver failure (ALF) (see further, the King’s College criteria for poor prognosis).

ALF is defined as ALI plus hepatic encephalopathy (HE) due to a primary liver insult. HE is based on clinical signs and psychometric or neurophysiological alteration of tests exploring psychomotor speed/executive functions and has five degrees [27]:

Minimal alteration of the tests (without clinical mental change)

Grade I: trivial lack of awareness/euphoria, or anxiety/shortened attention span/impairment of addition or subtraction/altered sleep rhythm, and clinical signs: mild asterixis or tremor.

Grade II lethargy or apathy/disorientation/obvious personality change/inappropriate behavior and clinical signs: obvious asterixis, dyspraxia, slurred speech.

Grade III somnolence to semi-stupor, responsive to stimuli/confusion/gross disorientation/bizarre behavior, and clinical signs: muscular rigidity, clonus, hyperreflexia.

Grade IV coma and clinical signs: decerebrate posturing.

The King’s College criteria for poor prognosis in non-paracetamol-induced ALF are either INR > 6.5, as a single criterion, or three out of five of the following criteria: (1) indeterminate hepatitis, drug-induced hepatitis; (2) age< 10 years or >40 years; (3) interval jaundice–encephalopathy >7 days; (4) bilirubin > 300 micromole/l (>18 mg/dl); (5) INR > 3.5.

The ultrasound examination of the liver considers hepatomegaly to be any craniocaudal dimension of the right lobe, measured on the midclavicular line, of more than 15 cm.

Statistical analysis of the parameters’ investigation was conducted using IBM SPSS Statistics 26.0.

Ethical: The study has the approval of the National Institute for Infectious Diseases Ethics Committee, Bucharest, Romania (C12229/2022). All patients provided written informed consent. Data supporting reported results can be found in the National Institute for Infectious Diseases ‘Matei Bals’ electronic database and archives.

## 3. Results

### 3.1. Demographic Features and Sexual Behavior

Fifty-one cases of acute hepatitis A were admitted in the first seven months of 2022.

The median age of all cases of hepatitis A was 27 years (between 3 and 58 years). Eight patients (n = 8/51, 15.68%) were children under 14 (one girl and seven boys).

The male-to-female ratio was 6.29:1 (44:7).

Thirty-seven of all fifty-one patients were young and adult males (n = 37/51, 72.54%). The median age of this male subgroup was 30 years, ranging between 17 and 58 years. Twenty (n = 20/37, 54.5%) had been identified as MSM.

Both groups, non-MSM (n = 23) and the MSM (n = 20), consisted of individuals of relatively young age. The non-MSM group had a mean of 27.69 years (range 37, minimum = 16, maximum = 53), a median of 26 years [IQR = 12], while the MSM group had a mean age of 33.2 years (range 40, minimum18, maximum 58), and a median of 32.5 [IQR = 11.5].

Sexual behaviors are presented in Table 1.

### 3.2. Sexually Transmitted Diseases and/or Other Acquired Coinfections

#### 3.2.1. History of STD and Concomitant STD

When we looked for previously diagnosed STDs, we found that 22 (n = 22/37, 59.45%) out of all males had concomitant STD or a history of STDs. There were in total 18 syphilis cases, of which 7 cases were of syphilis alone, and among them, 3 were primary syphilis concomitantly diagnosed with hepatitis A. Ten cases (n = 10/22, 10.8%) had both syphilis and HIV. In addition, one HIV and syphilis seropositive patient developed subsequent Mpox. There were also four cases with HIV (n = 4/22, 10.8%) alone. No woman had been diagnosed with previous or concomitant STDs. The number of cases is illustrated in Table 2.

#### 3.2.2. Concomitant Positive Serology for Hepatitis E Virus (Screening Tests)

Of the 43 cases of hepatitis A in adolescent and adult patients, 29 were tested for HEV-IgM, resulting in 15 negative and 14 positive cases. The presence of HEV-IgM showed no correlation with age, gender, sexual orientation, HIV status, or syphilis seropositivity in patients with hepatitis A. There was also no correlation with the severity of hepatitis or a poor outcome.

Confirmatory testing for co-existing acute hepatitis E was carried out in only four cases: three using HEV-RNA and one using HEV-IgG. All confirmatory tests were performed after a delay relative to the peak transaminase levels. The results revealed undetectable HEV-RNA in all cases, while the antibody-based test (HEV-IgG) was strongly positive.

### 3.3. Clinical and Biological Findings

All patients presented with ALI.

HE: No psychometric tests were performed. As for HE clinical signs, one patient had grade I HE and fulfilled the ALF criteria. None had scores indicating grades II-IV of HE.

Regarding ALI, all 43 patients had elevated hepatic enzymes and bilirubin levels, with a median of 2731 U/L for ALT and a median of 6 mg/dl for direct bilirubin level. In addition, we divided the patients into two groups: non-MSM and MSM patients. When the median levels for ALT and direct bilirubin were compared between these two groups, the MSM group had a higher mean level: the ALT median level was 3404 U/L [IQR 2608–5448] in the MSM group vs. 2066 U/L [IQR 1393–3682] in the non-MSM group (*p*-value 0.034). The direct bilirubin median level was 7.4 mg/dl [IQR 4.4–9] in the MSM group vs. 5.5 mg/dl [IQR 3.7–7.5] in the non-MSM group, respectively (*p*-value 0.185).

Coagulation parameters were also compared between these two groups. We found a median INR level of 1.37 in the MSM group [IQR 1.18–1.78] and 1.18 [IQR 1.11–1.43] in the non-MSM group, respectively (*p*-value 0.040). Regarding prothrombin concentration in the MSM group, the median level was 60.3% [IQR 43.8–75] vs. 75.5% [IQR 56.67–81.92] in the non-MSM group (*p*-value 0.024). Significant coagulation disturbances (prothrombin concentration < 30%, INR > 2.2) were present exclusively in the MSM group, in four cases. All the biological findings are presented in Table 3.

### 3.4. Ultrasound Examination Findings

All patients had hepatomegaly, and none had a shrinking liver volume during the disease course.

### 3.5. Outcome

All 43 cases had a favorable outcome under standard symptomatic and/or pathogenic therapy. Patients with concomitant syphilis diagnosis received appropriate antibiotic therapy. No one needed intensive care, and they were discharged after a median period of 8 days.

## 4. Discussion

### 4.1. Post-Pandemic Outbreak in Romania Among Other European Countries

During the pandemic, a decline in cases was observed; this was likely attributable to the implementation of social distancing measures. However, as these measures were relaxed, a sharp resurgence in cases emerged at the beginning of 2022. Romania was significantly affected, ranking third among EU/EEA countries regarding notification rate, with 4.8 cases per 100,000 population, and taking the lead in total reported cases, with 917 [28].

From the same Annual Epidemiological Report 2022, from the ECDC, 30 EU/EEA countries reported 4548 cases of hepatitis A, and the notification rate for the entire EU/EEA was one case per 100.000 population. Children between the ages of 5 and 14 still represent the largest category of cases (20%) among the total of 4548 cases. But some particularities were described. For example, Hungary (which had the highest notification rate, of 5.5 per 100.000 population) reported over 160 cases between December 2021 and September 2022, and 86% of the patients were males [28]. A paper published by Dencs et al. in 2024 [29] analyzed samples from 224 HAV IgM-positive patients diagnosed in 2022 in Hungary, and the conclusion was that a unique subtype IB virus was detected in most samples. Epidemiological data sustained the hypothesis that sexual transmission had been driving the outbreak because of the high male-to-female ratio and the significant number of patients who had other sexually transmitted infections, including HIV [29]. The authors also linked the outbreak to a frozen berry source originating from a restaurant [29]. Another paper from Croatia reported a hepatitis A outbreak among the MSM population using pre-exposure prophylaxis (PrEP) and people living with HIV. A total of 77 cases were reported, with most cases being diagnosed in mid-February 2022. The HAV subtype IA was detected in all cases where sequencing was available [30]. Maybe it is worth noting that Croatia had the second highest notification rate in the EU/EEA region in 2022 [28]. Most cases were reported among males aged between 25 and 44 and occurred in the first four months of 2022 [28,30].

Returning to Romania, from the ECDC 2022 report, children under 14 accounted for two cases per 100,000 population, representing roughly 40% of all reported cases in Romania for that year [28].

In contrast, in our setting, which is one of the two largest Romanian infectious disease centers but also the largest Romanian center for HIV/AIDS treatment and care, the percentage of children in the first seven months after the pandemic was small (16%), and instead, there was a large majority of young MSM affected.

In line with our findings, our colleagues from the other largest tertiary infectious diseases hospital in Romania, and the second largest Romanian center for HIV/AIDS treatment and care (Victor Babes Hospital, Bucharest), conducted a study that reported 35 cases of hepatitis A in the MSM population during the first half of 2022 [31].

Our colleagues from Victor Babes Hospital [31] found no significant differences in clinical outcomes between HIV-infected and HIV-negative patients. This observation may hold in our setting as well, since we had favorable outcomes for all cases of hepatitis A, irrespective of sexual behavior or HIV status. However, regarding the severity of hepatitis A (the degree of liver dysfunction during the acute phase, ALI, and coagulopathy, respectively), we found significant differences between groups; in the MSM group, the hepatitis A severity was significantly higher. Although there are authors who have concluded that MSM status and HIV infection do not influence the clinical course or severity of hepatitis A [32], others highlight the increased severity of hepatitis A within the MSM population [20,21,22,23,24], as per our actual study.

### 4.2. Sexually Linked Hepatitis A—Co-Existence with Other STDs

Sexually linked hepatitis A acquisition involves genito/ano-oral intercourse between a contagious person and a naïve, non-immunized person. An individual becomes contagious before symptoms appear, making HAV more easily circulate among “apparently” healthy humans [33]. Since 1996, the Advisory Committee on Immunization Practices (ACIP) has recommended vaccination against hepatitis A for MSM individuals [34]. Although the vaccine confers protection with an efficacy above 95% for up to 15 years in healthy persons, in the HIV population, the immune response can be weaker, with lower protection rates, depending on the degree of immunosuppression [35]. As mentioned above, the Romanian national program for vaccination does not include vaccination against hepatitis A.

In our group of patients, we also found other conditions related to risky sexual behavior: two-thirds of male patients had STDs, and more than half were previously diagnosed with syphilis, HIV, or both HIV and syphilis.

Moreover, one HIV-infected patient, with a past syphilis infection, developed Mpox in the next two months after being diagnosed with hepatitis A.

The double acute concomitant infection, hepatitis A and primary syphilis, was noted in three males (8.1%) in our study group, exclusively in young men. As hepatitis A and primary syphilis have roughly similar incubation periods (around three weeks), we can speculate that both illnesses were acquired at the same time.

This pattern, with over half of the young males with hepatitis A having previous or concomitant STDs, highlights the importance of hepatitis A transmission linked to sexual practices between MSM individuals.

Other studies are in line with our findings, reporting a high incidence of coinfection with other sexually transmitted infections. For example, Boucher et al. reported that at least one concomitant STD was diagnosed in 54% of tested patients with hepatitis A, with a high rate of hospitalization [23].

It is also essential to identify the factors that predispose MSM patients to more severe hepatitis A-related liver injury, as observed in our study. One plausible explanation is that sexual practices within this population may lead to exposure to a higher viral inoculum, potentially contributing to the severity of liver injury. Additionally, the association with preexisting or concomitant sexually transmitted diseases warrants further investigation as a potential trigger for a more severe course of hepatitis A.

### 4.3. Coinfection Hepatitis A Plus Hepatitis E—A Secondary Possible Finding in Our Study

Although they share the common fecal–oral transmission routes, and outbreaks of hepatitis A in the MSM populations have been widely reported, HEV infection through person-to-person contact is rare [36,37]. In the MSM population, HEV has a low rate of transmission even during hepatitis A outbreaks [38,39,40,41], and sexual transmission does not seem to be a significant route of HEV infection. But a study published by Greco et al. noted that the rate of coinfection HEV/HAV was 14.6% in MSM and 1% in the control group, suggesting that HEV should be tested during hepatitis A outbreaks in MSM populations [42].

In our study, almost one-third of patients were tested with HEV-IgM, and interestingly, we found that nearly half of them were IgM-seropositive. In addition to HEV-IgM, HEV-RNA or HEV-IgG testing is required for diagnostic confirmation. However, we were only able to perform these tests sporadically. Among the 14 positive cases, HEV-RNA testing was conducted in 3 instances, but serum sampling occurred well after the peak transaminase levels (due to delayed HEV-IgM results). Consequently, all HEV-RNA results were negative, which might indicate that we did not collect the serum in time, preventing us from detecting the virus during its short viremia period, or the HEV-IgM tests yielded false-positive results. HEV-IgG testing was performed in one case, and the result was positive. The severity and outcome of hepatitis A were not influenced by the presence, whether real or false, of concomitant hepatitis E in our group. Additionally, no correlation was found between HEV-IgM and age, gender, sexual orientation, HIV status, or syphilis seropositivity in our study group. However, this observation warrants further investigation.

### 4.4. The Assessment of Viral Hepatitis Severity—The Need for Adapting the Severity Scores

The EASL criteria for ALI in acute viral hepatitis are not well-suited for this context (specifically viral hepatitis), nor are the HE criteria (see the Methods Section). For instance, in any acute viral hepatitis, liver enzyme elevations exceeding ten times the normal value are a defining feature. Thus, the ALI definition of enzyme elevations at two to three times the normal values fails to differentiate among cases of acute viral hepatitis.

Furthermore, total and direct bilirubin levels are commonly highly elevated in over 70% of adults with viral hepatitis, while more than 70% of children remain asymptomatic. The single cut-off of 18 mg/dL for direct bilirubin, as per the King’s College criteria for poor prognosis, lacks applicability without further differentiation.

INR is frequently elevated in acute viral hepatitis, with cut-offs of 3.5 and 6.5 potentially indicating poor and very poor prognoses, respectively.

The presence or absence of HE should be actively assessed in cases of acute viral hepatitis. Although arterial ammonia is not part of the routine laboratory panel in acute hepatitis, it might hold value in establishing objective criteria for mild HE.

In our study, the degree of liver injury differed significantly between the MSM and non-MSM groups. One patient met the criteria for ALF by presenting a grade I HE. However, none of the cases were actively assessed for minimal HE abnormalities. Regarding the King’s College criteria [26] for poor prognosis in ALF, none of the patients in our cohort met these criteria. Moreover, no patient experienced life-threatening complications or required intensive care. All cases had favorable outcomes, with a median hospital stay of eight days before discharge.

### 4.5. Study Limitations

Our analysis is retrospective, covering the first seven months of 2022 rather than the entire year, and focuses on a single Romanian setting, despite it being the largest tertiary infectious diseases hospital. Of the 900 Romanian cases reported to the ECDC in 2022, our study accounted for approximately 5%.

Due to the retrospective nature of our research and other factors, certain key aspects were not thoroughly assessed, such as liver function in relation to HE scores.

A significant limitation of our study is the absence of molecular analysis to confirm transmission among MSM or to trace our cases within broader European outbreaks.

Additionally, we could not establish concomitant hepatitis E within our study group. Coinfection of hepatitis A and E was confirmed in only one case, the sole patient tested for HEV-IgG. In the remaining 13 cases, coinfection remains speculative.

### 4.6. Future Research Directions

We are interested in ‘tracking’ new outbreaks. As derived from the previous subsections, further research is needed on the interrelation between hepatitis A and E, as well as STDs, and in establishing and implementing proper severity scores and prognosis in acute viral hepatitis. Moreover, there is a need to reinforce the vaccination program to mitigate preventable diseases.

## 5. Conclusions

Our findings suggest that the Romanian hepatitis A outbreak emerged at the beginning of 2022 because of the relaxation of COVID-19 pandemic measures, as did other hepatitis A outbreaks in Europe and the world. Its particularity is that many cases were linked to the at-risk sexual behavior—most of the patients were MSM and had previously or concomitant STDs like syphilis and HIV. Therefore, when diagnosing hepatitis A, especially in a young male patient, it is advisable to search for sexual behavior and STDs. Moreover, it is of interest to establish whether MSM-linked transmission and/or the presence of STDs are predisposing factors to a more severe hepatitis A course. Based on our findings, we believe that it is essential to define more specific severity criteria regarding viral hepatitis. We also highly recommend a vaccination-reinforcing policy, with the inclusion of the hepatitis A vaccine in the national vaccination program, targeting individuals with high-risk sexual behaviors from the start.

## Figures and Tables

**Table 1:** Adolescent and adult patients with hepatitis A (n = 43 patients): gender distribution and sexual behavior-related risk.

Female n = 6	Male n = 37		
No sexual behavior risk declared	No sexual behavior risk declared	High-risk sexual behavior declared	MSM declared
**6**	**16**	**1** USC	**20**

Legend: USC = unprotected sexual contact without mention of the MSM status, MSM = men who have sex with men.

**Table 2:** History of STD and concomitant STD at the Hepatitis A diagnosis.

Syphilis	HIV	Syphilis + HIV	Syphilis + HIV + Mpox
**7** History or secondary syphilis in 4; primary syphilis in 3	**4** HIV (+) status known	**10** HIV (+) and syphilis (+) status known	**1** HIV (+) and syphilis (+) statusknown; subsequent Mpox

Legend: STD—sexually transmitted diseases.

**Table 3:** Biological findings in the non-MSM group versus the MSM group.

**Lab Findings**	**Non-MSM Group**	**MSM Group**	***p*-Value**
**ALT** (U/L) (range)	**2066** (304–7269)	**3404** (444–12,190)	**0.034**
**D Bilirubin** (mg/dl) (range)	**5.5** (0.8–16.6)	**7.4** (0.6–16)	0.185
**INR** (range)	**1.18** (0.9–2.28)	**1.37** (1.02–4.24)	**0.040**
**Prothrombin (%)** (range)	**75.5** (31.6–113)	**60.3** (16–96)	**0.024**

Legend: ALT–alanine aminotransferase; D Bilirubin—direct bilirubin; INR—International Normalized Ratio; Prothrombin (%)—prothrombin concentration. All values are expressed as median and their range in brackets; MSM—men who have sex with men.

## Data Availability

The data presented in this article are available upon request from the corresponding authors. The raw patients’ data are preserved in the electronic database of the National Institute for Infectious Diseases Matei Bals, Bucharest, Romania, and consist of their files (diagnosis, history, outcome, and all laboratory tests). Our institute’s database has contained patients’ data since 2000.

## Abbreviations

The following abbreviations are used in this manuscript:

ALI	Acute liver injury
ALF	Acute liver failure
ALT	Alanine-transferase
EU/EEA	European Union/European Economic Area
HA	Hepatitis A
HAV	Hepatitis A virus
HEV	Hepatitis E virus
HE	Hepatic encephalopathy
HIV	Human deficiency virus
MSM	Men who have sex with men
PrEP	Pre-exposure prophylaxis
STD	Sexually transmitted disease
